# Genomic signatures of G-protein-coupled receptor expansions reveal functional transitions in the evolution of cephalopod signal transduction

**DOI:** 10.1098/rspb.2018.2929

**Published:** 2019-02-27

**Authors:** Elena A. Ritschard, Robert R. Fitak, Oleg Simakov, Sönke Johnsen

**Affiliations:** 1 Department of Molecular Evolution and Development, University of Vienna, Vienna, Austria; 2 Department of Biology, Duke University, Durham, NC, USA

**Keywords:** G-protein-coupled receptors, cephalopod evolution, gene family expansion, gene duplication, signal transduction

## Abstract

Coleoid cephalopods show unique morphological and neural novelties, such as arms with tactile and chemosensory suckers and a large complex nervous system. The evolution of such cephalopod novelties has been attributed at a genomic level to independent gene family expansions, yet the exact association and the evolutionary timing remain unclear. In the octopus genome, one such expansion occurred in the G-protein-coupled receptors (GPCRs) repertoire, a superfamily of proteins that mediate signal transduction. Here, we assessed the evolutionary history of this expansion and its relationship with cephalopod novelties. Using phylogenetic analyses, at least two cephalopod- and two octopus-specific GPCR expansions were identified. Signatures of positive selection were analysed within the four groups, and the locations of these sequences in the *Octopus bimaculoides* genome were inspected. Additionally, the expression profiles of cephalopod GPCRs across various tissues were extracted from available transcriptomic data. Our results reveal the evolutionary history of cephalopod GPCRs. Unexpanded cephalopod GPCRs shared with other bilaterians were found to be mainly nervous tissue specific. By contrast, duplications that are shared between octopus and the bobtail squid or specific to the octopus' lineage generated copies with divergent expression patterns devoted to tissues outside of the brain. The acquisition of novel expression domains was accompanied by gene order rearrangement through either translocation or duplication and gene loss. Lastly, expansions showed signs of positive selection and some were found to form tandem clusters with shared conserved expression profiles in cephalopod innovations such as the axial nerve cord. Altogether, our results contribute to the understanding of the molecular and evolutionary history of signal transduction and provide insights into the role of this expansion during the emergence of cephalopod novelties and/or adaptations.

## 1. Introduction

G-protein-coupled receptors (GPCRs) comprise a large superfamily of membrane proteins that possess a characteristic 7-transmembrane domain (7TM) [1]. They trigger signal transduction pathways by sensing a variety of external and internal stimuli, including hydrogen and Ca^2+^ ions, small peptides (such as hormones and neurotransmitters), large proteins, and even photons as in the case of the light-sensitive opsins [2]. GPCRs thus play a crucial role in how organisms perceive and react to their environments, as well as in homeostatic regulation via endocrine and neuronal functions.

GPCRs have an ancient eukaryotic origin [3]. In bilaterians, the superfamily has evolved into five structural families: Glutamate, Frizzled, Adhesion, Secretin, and Rhodopsin [4]. Amino acid binding (e.g., glutamate and gamma-aminobutyric acid, GABA) is the predominant function in the members of the Glutamate family, but its expansion in vertebrates has resulted in pheromone-, taste, and calcium-sensing functions, as well as olfaction in fishes [5]. Receptors of the Frizzled family have a crucial role in animal development and therefore represent the most conserved family among GPCRs. Members of the Adhesion family are characterized by long N-termini that can contain several domains with adhesive properties (hence the ‘adhesion’ nomenclature), resembling or complementing the function of other molecules such as cadherins or integrins [6,7]. The proper processing and activation of the Adhesion GPCRs are thought to depend upon their conserved domain, the GPCR proteolytic site (GPS), but their exact physiological function remains unknown [8]. The Secretin family is closely related to the Adhesion GPCRs [5] and represents one of the largest groups of hormone and neuropeptide receptors involved in homeostatic functions [9]. Lastly, the Rhodopsin family is the largest group of GPCRs in most animals. Its members bind chemically diverse ligands and—in certain cases—sense light, thus mediating a variety of functions such as vision, neurotransmission, and immune responses.

Despite the conservation of the GPCR families during bilaterian evolution, the number of receptors in each family varies within individual lineages [3]. The enlargement of GPCR repertoires predominantly occurs by gene duplication and subsequent independent evolution of the copies [5], a powerful evolutionary mechanism that generates novel functions (via e.g. neo- or subfunctionalization) [10,11]. GPCR expansions are not surprising considering the diverse environments and stimuli these receptors evolved to process. Larger repertoires can increase the amount of available sensory information, thus facilitating adaptation to the environment [5] by enabling the evolution of sensory functions relevant to ecological contexts of species (e.g. gustatory receptors in disease-transmitting mosquitoes [12], chemoreceptors in nematodes [13,14], olfactory receptors in mammals [15]). Moreover, such expansions permit more complex homeostatic regulation [16]. However, relatively little is known about GPCR diversity and functionality outside vertebrates and model invertebrate species (e.g. *Drosophila melanogaster* [1]).

The cephalopod (referring in this paper to coleoid cephalopods) body plan and nervous system are unique among molluscs. Morphological and neural novelties in these animals include flexible arms and a large and complex nervous system that may reach a total of 500 million neurons [17,18]. Rivalling vertebrate nervous systems, it is considered the largest among invertebrates. The cephalopod nervous system consists of central lobes surrounding the oesophagus and two optic lobes that together contain approximately a third of the neurons, with the remaining two-thirds distributed within the arms (e.g. in the axial nerve cord) [19]. This nervous system controls an outstanding behavioural repertoire [20], including their camouflaging abilities. Chromatophore cells contain pigment granules that are contracted or expanded through adjacent muscle action, allowing the animals to rapidly adjust colouration [21]. Moreover, cephalopod arms are considered a key innovation for their diversification as they might have enabled these animals to become agile predators [22]. The arms bear hundreds of tactile and chemosensory structures, known as suckers that interact with and provide information from the environment [23]. These structures, as well as the muscle coordination of the arms, are controlled by the axial nerve cords [19]. Additional perception of the external environment is achieved by the convergently evolved camera-type eyes harbouring one photoreceptor type and potentially by dermal photoreception [24].

Insights into the molecular basis of cephalopod innovations first arose with the sequencing of the California two-spot octopus' (*Octopus bimaculoides*) genome [25]. The genomic analyses revealed expansions in several key gene families involved in neuronal patterning, such as C2H2 zinc fingers and protocadherins, and additionally the GPCRs. The individual family composition, evolutionary dynamics, and patterns of gene expression of this octopus GPCR expansion, however, have not been studied yet. Therefore, our aim was to assess the evolutionary history of this expansion and its relationship with morphological and neural cephalopod novelties. We studied genomic signatures of this gene superfamily in cephalopods, revealing potential evolutionary mechanisms behind their expansion and expression. Our results provide information about the duplication dynamics in this superfamily of proteins and the evolution of GPCR-mediated signal transduction in cephalopods.

## 2. Material and methods

### (a) Data collection and phylogenetic analyses

Pfam signatures for the Rhodopsin family (also classified and found in the Pfam database as class A: pf00001), Secretin and Adhesion families (both under class B: pf00002), Glutamate family (class C: pf00003), and Frizzled family (class F: pf01534) were used to compile a dataset of protein sequences from the UniProt database [26] for 14 species: *Anopheles gambiae* (African malaria mosquito), *Drosophila melanogaster* (fruit fly), *Caenorhabditis elegans* (round worm), *Capitella teleta* (polychaete worm), *Helobdella robusta* (Californian leech), *Schistosoma mansoni* (blood fluke), *Strongylocentrotus purpuratus* (purple sea urchin), *Saccoglossus kowalevskii* (acorn worm), *Branchiostoma floridae* (Florida lancelet), human*, Lottia gigantea* (giant owl limpet), *Mizuhopecten yessoensis* (Japanese scallop), *Crassostrea gigas* (Pacific oyster), and *O. bimaculoides* (California two-spot octopus). If available, only entries matching a reference UniProt proteome were downloaded (see electronic supplementary material, table S1). *Euprymna scolopes* (Hawaiian bobtail squid) and *Callistoctopus minor* (common long-arm octopus) sequences corresponding to the same GPCR families were included in the study using draft genome sequences (*E. scolopes:* Belcaid *et al*. [27]; *C. minor:* Kim *et al*. [28]). These 16 species were selected for our analysis to reconstruct the history of the cephalopod GPCR repertoire under a broad evolutionary context, having points of comparison between cephalopods (i.e. two Octopodiformes and one Decapodiformes species) and other species covering major bilaterian lineages (electronic supplementary material, figure S1). After removing protein sequences corresponding to equal gene entries (i.e. isoforms), the final dataset of all GPCRs comprised a total of 6194 sequences*.* Individual datasets for each GPCR class (A, B, C, and F) were also constructed for the phylogenetic analyses (electronic supplementary material, table S1). Sequences were aligned with MAFFT v7.312 [29] (default parameters) and alignments were cleaned with TrimAl v1.4 [30] using a 0.25 gap threshold, 0.25 residue overlap threshold, and 90% sequence overlap. The best-fit model of molecular evolution for each dataset was selected with ModelFinder, implemented in IQ-TREE v. 1.6.2 [31] using the corrected Akaike Information Criterion (AICc). For the class A, class F, and all GPCRs datasets, the LG model was assigned [32]. For class B and C, the WAG model was selected [33]. Maximum-likelihood trees were constructed with FastTree v2.1.10 [34] using four rounds of minimum-evolution nearest neighbour interchanges (NNI) and remaining parameters as default. Local support values were computed with FastTree by default with the Shimodaira–Hasegawa (S–H) test, a test that compares multiple topologies based on a non-parametric bootstrap [35,36]. To identify the expanded clades of GPCRs, we first identified nodes containing only cephalopod GPCRs (sequences of only one, two, or three cephalopod species) in the full GPCR tree and then ranked each node by the number of cephalopod GPCR sequences descending from the node. We only retained nodes that were outliers (10 or more paralogues) in the number of GPCRs present as expanded groups (electronic supplementary material, figure S2). Files with the sequences, alignments, and trees are available from the Dryad Digital Repository: https://doi.org/10.5061/dryad.d3qh5c8 [37].

### (b) Gene expression analyses

Normalized expression data, measured as transcripts per total expression counts, were collected across 12 tissue types for the genes encoding the *O. bimaculoides* GPCRs using a publicly available dataset [25]. Gene expression data, normalized as TPM counts (Transcripts per Million), for GPCRs of *C. minor* were collected across 18 tissue types from [28]. Expression data (TPM) for *E. scolopes* GPCRs were also collected across 7 tissues [27]. Tissue specificity of genes was measured calculating the parameter Tau [38], which varies between 0 (broad expression) and 1 (tissue specific). Genes with a Tau value higher than 0.8 were considered tissue specific [39]. Tau values between the non-expanded and the expanded groups were compared with a Kruskal–Wallis rank test [40]. A Dunn's test [41] was performed to report the results among the pairwise comparisons, using the Holm's *p*-value adjustment method [42]. Additionally, the proportion of expression of each gene in the different tissues was calculated by dividing its expression value in a tissue by the sum of its expression values in all tissues. The expression matrices for *E. scolopes*, *O. bimaculoides*, and *C. minor* used for the aforementioned analyses and calculations performed were deposited in Dryad Digital Repository: https://doi.org/10.5061/dryad.d3qh5c8 [37].

### (c) Annotation of expanded GPCR groups

Annotation of the nearest sequences to the expanded groups in the individual class trees was used to infer the putative function of the recently duplicated cephalopod GPCRs. Lastly, *O. bimaculoides* and *C. minor* GPCR sequences from the expanded groups were assigned putative functions by scanning for matches against protein signatures from the InterPro member databases [43] using InterProScan v5.7.48 [44].

### (d) Positive selection analysis and GPCR genome location

Expanded groups were examined for positive selection by performing a maximum-likelihood branch test with codeml in PAML v4.9 [45]. Following the tree topology from the individual class phylogenies, subsets of the trees corresponding to the node containing each expanded group and its outgroup (S–H support greater than 0.70) were extracted using the drop.tip function in R (ape v5.0 package [46]). Protein sequences were re-aligned with MAFFT and resulting alignments were converted to their corresponding coding DNA sequence (CDS) alignments using the PAL2NAL program [47]. The CDS alignment was then cleaned with TrimAl, erasing sequences with not enough informative sites by using a 0.25 residue overlap threshold and 90% sequence overlap. In the case of *O. bimaculoides, C. gigas*, and *L. gigantea*, CDS sequences were obtained from the Ensembl Metazoa genomes browser (https://metazoa.ensembl.org/index.html) [48]. *C. minor* sequences were obtained from the supporting data of Kim *et al*. [28]. For the remaining species (i.e. *A. gambiae*, *D. melanogaster*, *M. yessoensis*, *S. purpuratus*, *B. floridae*, and human), corresponding coding DNA sequences were downloaded from NCBI (National Center for Biotechnology Information), searching by the gene ID entries. For each group, the CDS alignment and tree topology were used in codeml to compare likelihoods from two different models: (i) a null model assuming a single ratio of non-synonymous to synonymous substitutions (*ω*; calculated by codeml from the dataset) across the entire tree, and (ii) an alternative model that estimated a different *ω* in the branches of interest (foreground), relative to the remaining (background) branches. For the alternative model, branches within groups 1–4 were labelled as foreground, whereas all other branches remained as background. The null and alternative models were compared with a likelihood ratio test, where the test statistic is chi-squared distributed with *k* degrees of freedom (*k* being the difference in the number of free parameters between models). Additionally, a sites test was carried out to identify specific amino acids under positive selection. In this test, the same null model was compared to an alternative model that allowed *ω* to vary among both branches and amino acid sites. Sites with a Bayes empirical Bayes (BEB) confidence greater than or equal to 95% and *ω* > 1 were identified as under positive selection [49,50]. In the branch test, branches with *ω* > 100 were discarded because they often overestimate *ω* as a result of poor alignment quality between deeply split or otherwise highly divergent sequences. Input files and codeml outputs, as well as likelihood ratio test calculations were deposited in Dryad Digital Repository: https://doi.org/10.5061/dryad.d3qh5c8 [37].

Lastly, to search for evidence of tandem duplication, scaffold locations of *O. bimaculoides* genes from both non-expanded and expanded groups were extracted from Albertin *et al*. [25]. Start and stop positions in the scaffolds, as well as the direction of these genes, were summarized and can be found in electronic supplementary material, table S2.

## 3. Results

### (a) Phylogenetic analysis

**Figure 1.**
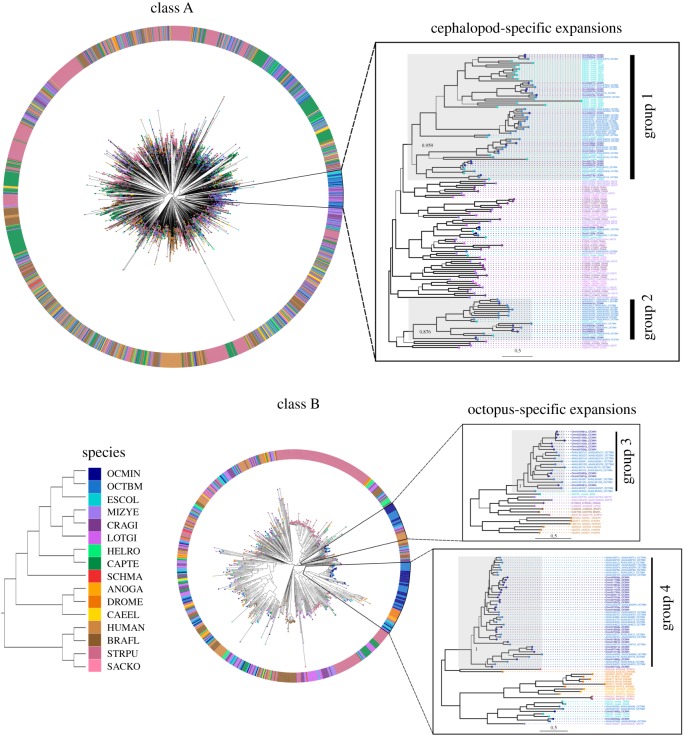
Phylogenetic trees for class A and B. Each colour corresponds to a species; abbreviations as follow. ANOGA: *Anopheles gambiae*, BRAFL: *Branchiostoma floridae*, CAEEL: *Caenorhabditis elegans*, CAPTE: *Capitella teleta*, CRAGI: *Crassostrea gigas*, DROME: *Drosophila melanogaster*, ESCOL: *Euprymna scolopes*, HELRO: *Helobdella robusta*, HUMAN, LOTGI: *Lottia gigantea*, MIZYE: *Mizuhopecten yessoensis*, OCMIN: *Callistoctopus minor*, OCTBM: *Octopus bimaculoides*, SACKO: *Saccoglossus kowalevskii*, SCHMA: *Schistosoma mansoni*, STRPU: *Strongylocentrotus purpuratus.* Zoom-ins show the expanded groups identified (1–4). Thicker branches represent significant S–H support (greater than 0.7). Bar length indicates 0.5 substitutions per site corresponding to approximately 250 Myr. Tree visualization was performed with the ggtree package [51] using R v3.4.2 [52]. (Online version in colour.)

The phylogenetic analyses resolved approximately 77% of branches at S–H support values greater than or equal to 0.70 in the tree containing all GPCR classes, 78% in class A, 83% in class C, and 79% in class B and F trees (see tree files deposited in Dryad Digital Repository). Using the ranking procedure of GPCR clades and a cut-off of at least 10 cephalopod sequences (Material and methods; electronic supplementary material, figure S2), two cephalopod-specific and two octopus-specific expansions were identified (labelled as groups 1–4 in figure 1) and distinguished from GPCRs shared with other bilaterians. Groups 1 and 2 belong to class A (Rhodopsin family) and contain, respectively, 63 and 21 GPCRs from the three cephalopod species, forming the cephalopod-specific expansions (S–H support greater than 0.70). Groups 3 and 4 belong to class B and contain 21 and 45 Secretin/Adhesion GPCR sequences from the two octopus species, respectively (i.e. *O. bimaculoides and C. minor*, with the exception of a single *E. scolopes* sequence in group 3; S–H support greater than 0.70). These constitute the putative octopus-specific expanded groups. We additionally estimated sequence divergence, based on total branch lengths, which resulted in a median of around 2.01 (first quartile 1.79, third quartile 2.5) substitutions per site between sequences in the expanded groups versus their closest non-cephalopod relatives. This indicates that, on average, around 1 substitution per site corresponds with the Cambrian radiation of those animal groups (approximately 500 million years ago) and 0.5 substitutions per site thus correspond with approximately 250 million years.

### (b) Gene expression analyses

**Figure 2.**
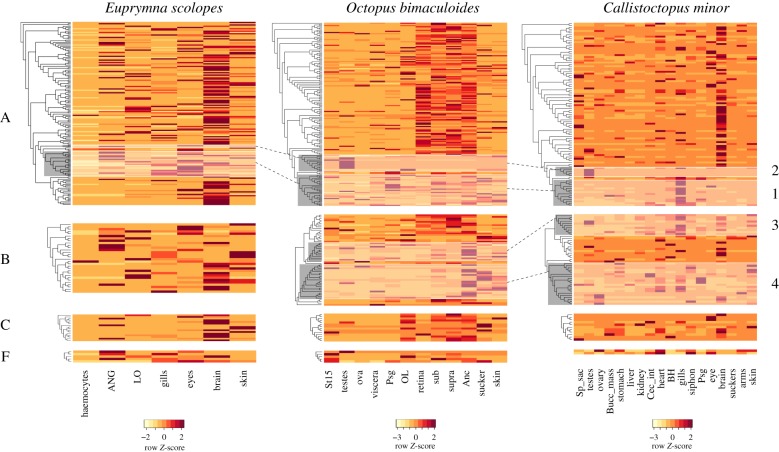
Heatmap of all *E. scolopes*, *O. bimaculoides*, and *C. minor* GPCRs. Genes (rows) are clustered following the trees resulting from the phylogenetic analyses performed for each class (*a*–*f*) independently. Clades highlighted in grey represent the four groups depicted in figure 1, group number is given to the right of the *C. minor* heatmap. Dotted lines connect corresponding expanded groups in both species. Tissues (columns) with transcriptomic data for *E. scolopes:* haemocytes, accessory nidamental gland, light organ (LO), gills, eyes, brain, and skin. Tissues (columns) with transcriptomic data for *O. bimaculoides*: sucker, testes, stage 15 (St15) embryo, ova, skin, posterior salivary gland (Psg), viscera (heart, kidney, and hepatopancreas), subesophageal brain (sub), supraesophageal brain (supra), optic lobe (OL), axial nerve cord (Anc), and retina. Tissues (columns) with transcriptomic data for *C. minor*: liver, kidney, stomach, caecum intestine (Cec_int), posterior salivary gland (Psg), buccal mass (Bucc_mass), bronchial heart (BH), systemic heart (heart), suckers, arms, skin, gills, siphon, brain, eye, spermatophore sac (Sp_sac), testes, and ovary. Heatmap generated with the heatmap.2 function (gplots v3.0.1 package [53]) using R v3.4.2 [52]. (Online version in colour.)

**Figure 3.**
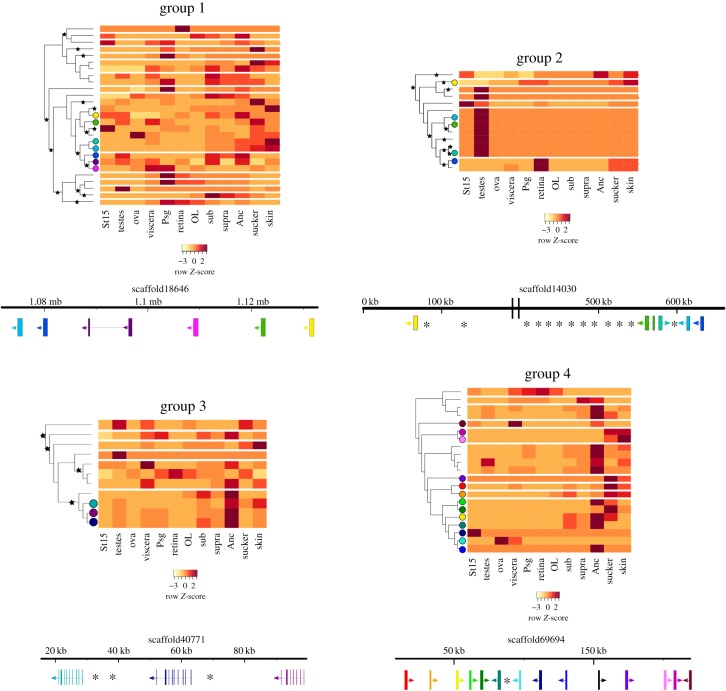
Expression profiles, positive selection results, and gene localization of cephalopod-specific expansions (groups 1 and 2) and octopus-specific expansions (groups 3 and 4) for *O. bimaculoides*. Genes (rows) in heatmaps are clustered following the trees resulting from the phylogenetic analyses. Spaces between rows indicate the presence of other sequences of cephalopod species as determined in the phylogenetic analyses. Black stars represent positive selection (*ω* > 1). The scaffold with the most co-localized genes per group is represented below the heatmaps (see detailed information in the electronic supplementary material, table S2). Exon–intron composition of the genes is depicted as thick bars (exons) and grey lines connecting them (introns). Direction of transcription is shown with an arrow. Asterisks (*) represent other genes (no GPCRs) found in the surrounding space of the co-localized GPCRs.

**Figure 4.**
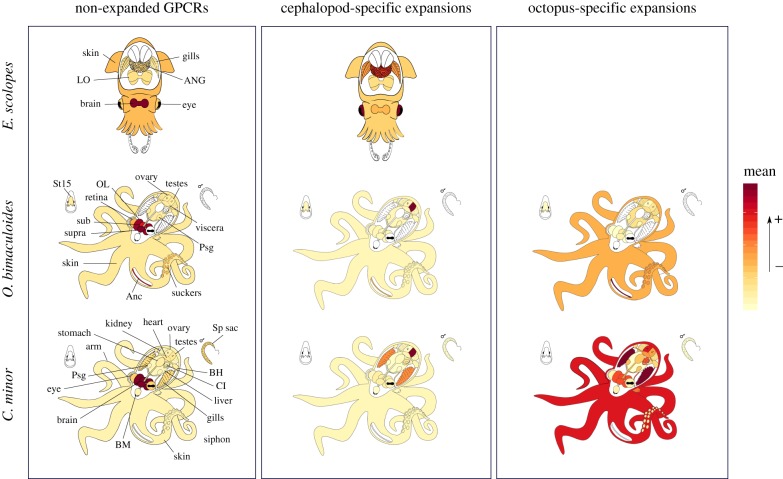
Expression proportions of *E. scolopes*, *O. bimaculoides*, and *C. minor* genes in each tissue in non-expanded GPCRs, cephalopod-specific expansions, and octopus-specific expansions. Tissue colouration gradient follows the mean values of gene expression proportion as shown in electronic supplementary material, figures S5–S7. Warmer colours represent the highest proportion of gene expression. *E. scolopes* (illustration designed by Hannah Schmidbaur) tissues*:* accessory nidamental gland (ANG), light organ (LO), gills, eyes, brain, and skin. *O. bimaculoides* tissues: sucker, testes, stage 15 (St15) embryo, ovary, skin, posterior salivary gland (Psg), viscera (heart, kidney, and hepatopancreas), subesophageal brain (sub), supraesophageal brain (supra), optic lobe (OL), axial nerve cord (Anc), and retina. *C. minor* tissues: liver, kidney, stomach, caecum intestine (CI), posterior salivary gland (Psg), buccal mass (BM), bronchial heart (BH), systemic heart (heart), suckers, arms, skin, gills, siphon, brain, eye, spermatophore sac (Sp sac), testes, and ovary. (Online version in colour.)

To explore the possible functions of the cephalopod GPCRs and evolutionary transitions between the identified expansions, the expression was examined across various tissue types (figures 2–4; electronic supplementary material, figures S5–S9 and table S2). A majority of non-expanded GPCRs (i.e. genes not included within groups 1–4) were highly expressed in the nervous tissues of *O. bimaculoides*, such as the sub- and supraoesophageal brain, optic lobe, and axial nerve cord, and the brain tissue in *C. minor* and *E. scolopes* (figures 2 and 4; electronic supplementary material, figures S5–S7). By contrast, expression of GPCRs in groups 1–4 was dominant outside of the brain (figures 2–4; electronic supplementary material, figures S5–S7). Expression in all groups was found to be mainly tissue specific. Mean Tau value and its 95% confidence interval in all but group 1 in *O. bimaculoides* and *E. scolopes*, and group 3 in *C. minor* was higher than 0.8 (electronic supplementary material, figure S4).

GPCR genes from the cephalopod-specific expansion had a large expression component devoted to the testes in both *C. minor* and *O. bimaculoides* (group 2 in figure 3; electronic supplementary material, figures S6, S8, and S9). The genes mostly contributing to this high expression in testes formed a clade in group 2 and shared the same profile (figure 3; electronic supplementary material, figure S6). The highest proportion of expression in group 1 sequences of *C. minor* was in the gills (electronic supplementary material, figures S6 and S9). Expression profiles of *O. bimaculoides* genes in this cephalopod-specific group did not follow a phylogenetic pattern (i.e. closely related sequences did not show a similar profile, figure 3), and their highest expression was devoted to nervous and non-nervous tissues (electronic supplementary material, figure S8). In *E. scolopes*, the highest proportion of expression was found in the eyes, the ANG, and gills (figure 4; electronic supplementary material, figure S5). However, due to the low number of tissues sampled in the bobtail squid in comparison to both octopuses, no conclusions were drawn for this species.

The largest proportion of expression in the octopus-specific expansions in *O. bimaculoides* was in the axial nerve cord, followed by the suckers and the skin (figure 4; electronic supplementary material, figure S8). As in group 1, *O. bimaculoides* genes contributing to the highest expression (axial nerve cord) were found to be forming clades in groups 3 and 4 (figure 3). For the case of *C. minor*, the highest expression proportion values were found in the gills, the ovary, and the skin (figure 4; electronic supplementary material, figure S9). The highest expression in gills was mainly found in group 3 and these similar profiles were found again shared within clades (electronic supplementary material, figure S6). By contrast, *C. minor* genes in group 4 did not share similar expression profiles.

### (c) Annotation of expanded GPCR groups

To further infer the putative function of the GPCR in groups 1–4, the annotation of the sister group sequences to the expansions were summarized (electronic supplementary material, table S3). Additionally, InterProScan annotation of the sequences from the four groups was performed to obtain information about putative functional domains. Groups 1 and 2 were found in a clade composed largely of molluscan sequences (i.e. *C. gigas*, *L. gigantea*, and *M. yessoensis*). These were found to be receptors of a variety of neuropeptides, such as Tachykinin-like peptides, Orexin (whose orthologue in deuterostomes is Allatotropin [54]), Cholecystokinin, Cephalotocin, Gonadotropin-releasing hormone (GnRH) II, Neuromedin-U, and others. Group 3 was found to be closely related to six human and two *M. yessoensis* adhesion GPCRs. All other related sequences (from *C. gigas*, *L. gigantea*, *S. purpuratus*, and *B. floridae*) were found to be uncharacterized proteins. The InterProScan searches confirmed the annotation of the cephalopod sequences to be adhesion GPCRs as it resulted in the identification of either the GPS motif and/or the adhesion 7TM domain in all cephalopod sequences in the group (with the exception of three *C. minor* sequences) (electronic supplementary material, table S3). Additional domains found in sequences of this expansion were the leucine-rich repeat (in 5 out of 22 sequences) and the Death domain (in one *O. bimaculoides* sequence). GPCRs from the octopus-specific expansion group 4 were found to be closely related to Methuselah and Methuselah-like proteins (a subgroup of Secretin GPCRs) from *D. melanogaster*, *A. gambiae*, and *M. yessoensis.* All octopus sequences in this group were found to have matches with Methuselah-like 7TM signatures in our InterProScan searches (with the exception of three *C. minor* sequences).

### (d) Positive selection analysis

For groups 1, 2, and 3, the likelihood ratio test rejected (*p* < 0.05) the null hypothesis of homogeneous evolution (equal *ω*) along the tree in favour of an alternative model of accelerated evolution (or positive selection) (electronic supplementary material, table S4). Signatures of positive selection (*ω* > 1) could be found in branches of these three groups (figure 3; electronic supplementary material, figures S5 and S6). For group 4, the test did not reject (*p* > 0.05) the null hypothesis, favouring a model of homogeneous omega values of 0.306 (less than 1, purifying selection) along the tree. Moreover, no specific amino acid sites could be identified under positive selection (BEB less than 95%) using the sites test for any of the expanded groups. Positive selection was found, among others, on branches leading to genes sharing similar expression patterns, such as a high expression in *O. bimaculoides’* axial nerve cord and *C. minor*'s gills (figure 3; electronic supplementary material, figure S6).

### (e) Genomic co-localization of GPCRs

To understand whether the more recently duplicated GPCRs tend to co-localize in the genome, we profiled their genomic locations in the draft genome assembly of *O. bimaculoides*. We found that only a single scaffold (out of 155) harboured three or more GPCRs from the non-expanded set, whereas groups 1–4 had the following distributions, respectively: 1 out of 17 scaffolds, 2 out of 6, 1 out of 9, and 2 out of 2 (electronic supplementary material, table S2). While impeded by the fragmented nature of the genome, comparison between the individual expansion groups and the non-expanded GPCRs shows almost complete re-distribution of the latter, supporting the scenario of local, tandem duplication and later dispersion.

The expression profiles of GPCRs co-localized in the genome of the cephalopod-specific GPCR expansions did not follow the same expression pattern, except for some of the genes in group 2 with the shared highest expression in testes (figure 3). By contrast, most of the genes in the octopus-specific expansion groups localized on the same scaffolds were highly expressed in the axial nerve cord (figure 3; electronic supplementary material, table S2). Moreover, evidence of local duplication under positive selection was found in some of these genes with shared expression patterns (i.e. group 4, Scaffold40771; figure 3).

## 4. Discussion

### (a) Non-expanded GPCRs are predominantly expressed in cephalopod nervous tissues

GPCRs are essential components of animal nervous systems as they mediate signal transduction by binding neurotransmitters [55]. Here, we found that the non-expanded GPCRs (i.e. those outside of the expanded groups 1–4) in the three cephalopod species are mainly expressed in neural tissues, such as the sub- and supraoesophageal brains, the optic lobes, and the axial nerve cords in *O. bimaculoides* and the brain in *C. minor* and *E. scolopes* (figure 2; electronic supplementary material, figures S7–S9). This suggests that a large proportion of the GPCR repertoire related to neural functions in octopuses derives from evolutionarily conserved families of GPCRs, most showing one-to-one orthology with other bilaterian species. These are possibly correlated with shared and highly conserved neuronal signalling functions [56] and not with cephalopod neuronal innovations. Rather, those innovations seem to be related to other large gene families such as C2H2 and protocadherins [25,57].

### (b) Expression divergence in the cephalopod-specific expansions

In contrast with the non-expanded GPCRs, we found a shift in expression patterns in the cephalopod-specific expansions comprising different nervous and non-nervous tissues, mainly outside the brain (figures 2–4). Expression of duplicates in group 2 was most prominent in testes (electronic supplementary material, figures S8 and S9), contributed by closely related genes (figure 3; electronic supplementary material, figure S6). Both *O. bimaculoides* and *C. minor* showed similar patterns. By contrast, sequences of group 1 showed discordant expression patterns between the three species analysed. Divergent expression patterns were found between *O. bimaculoides* duplicates, with the highest expression covering a wide range of tissues (figure 3; electronic supplementary material, figure S8), whereas *C. minor* showed higher expression in the gills (figure 3; electronic supplementary material, figures S6 and S9). This inconsistency in expression patterns could be an artefact resulting from differences in tissue sampling (e.g. *O. bimaculoides* lacks expression data for the gills) or a result of divergence time as most of these duplications have a coleoid cephalopod origin. Moreover, expression of these cephalopod-specific sequences in Decapodiformes (e.g. *Euprymna scolopes)* needs to be further investigated.

Both cephalopod-specific expansions were found inside a molluscan clade of neuropeptide receptors (figure 1; electronic supplementary material, table S3). These receptors and their ligands comprise families with a bilaterian origin and are known to control a variety of physiological processes like reproduction and sexual behaviour (e.g. GnRH), heart activity (e.g. cholecystokinin [58]), and food intake (e.g. Neuromedin-U, orexin/allatotropin) [54]. Here, we found evidence that part of this expanded repertoire of neuropeptide receptors (i.e. group 2) has converged in Octopodiformes in functions devoted to mainly two tissues. On the one hand, most sequences of group 2 were related to male reproduction through their testes-specific expression; however, their exact function in this tissue remains unknown. On the other hand, the majority of group 1 sequences in *C. minor* were expressed in the gills. These are potentially related to specific functions in this tissue (e.g. respiration, circulation, excretion), as they were found to be tissue specific (electronic supplementary material, figure S4). Additionally, we found signatures of positive selection in some gill-specific receptors (figure 3), suggesting that the maintenance of the copies and their expression domains was potentially advantageous for these animals. This GPCR expression could be related to circulatory or respiratory adaptations in cephalopods as highly active marine predators [59]. However, expression data of gills in *O. bimaculoides* and other cephalopod species would be needed to confirm this trend.

### (c) Evolution of octopus GPCR paralogues

Similar to cephalopod-specific GPCRs, the expression of the octopus-specific expansions was not related to the brain (figures 3 and 4). In *O. bimaculoides*, the highest proportion of expression was predominantly in the axial nerve cord, followed by the suckers and the skin (figure 4; electronic supplementary material, figure S8), whereas in *C. minor*, it was found to be again in gills (mostly in group 3), followed by the skin and ovaries (figure 4; electronic supplementary material, figure S9).

Proteins of octopus-specific group 3 were identified as adhesion GPCRs, as we found evidence for the presence of its characteristic domains: the GPS and the adhesion 7TM domains. Additional functional domains found here, like the leucine-rich repeat, have been also reported in the N-termini of human adhesion GPCRs [6]. Despite the small amount of information available on this GPCR family, expression data in vertebrates have suggested an important role in the central and peripheral nervous system [6]. As these proteins resemble or complement other adhesive molecules, such as cadherins, they could be involved in neuronal plasticity and axon guidance. The octopus genome revealed an expansion of protocadherins, highly expressed in nervous tissues and exceptionally enriched in the axial nerve cord and the optic lobes [25,57]. Consistently, some of these receptors showed higher expression in the axial nerve cord of *O. bimaculoides* (figure 3). These constitute a potential functional cluster, as they were co-localized and showed signatures of positive selection. Enrichment of these adhesion GPCRs in *O. bimaculoides* was also found in the suckers and skin, structures directly associated with the peripheral nervous system of cephalopods. Thus, this expanded subgroup of GPCRs could be complementing the function of the enlarged protocadherin repertoire reported in genomes of octopuses in these tissues. An axial nerve cord sample is missing from *C. minor* transcriptome sampling. However, the skin showed high expression of sequences from that group, whereas total arm and sucker tissues did not (figure 4; electronic supplementary material, figure S9). Most of the sequences were also found to be highly expressed in the gills in *C. minor* (absent in the available *O. bimaculoides* tissue sampling). Thus, a consistent tissue sampling for transcriptomic data would help elucidate whether GPCRs expanded in the Octopodiformes lineage have diverged in expression in individual species or followed a shared pattern related to octopus-specific novelties.

Our annotation results suggest that the second octopus-specific expansion (group 4) comprises Methuselah-like GPCRs. The *methuselah* gene and its 15 paralogues (*methusaelah-like 1–15*) were first described in *Drosophila* [60]. These constitute a gene family with an early metazoan origin that has gone through various events of extinctions (e.g. in vertebrates) and expansions (e.g. in insects) [61]. Little is known about the function of the 15 paralogues, but the *methuselah* gene has been widely studied in *Drosophila* and has been found to be related with stress response, lifespan, and embryonic development [62]. Here, we found that the octopus-specific expansion derives from the Methuselah-like 15 GPCR, one of the most ancient paralogues in *D. melanogaster*. The evidence of high co-localization found in this group suggests that this expansion arose from various events of tandem duplication (figure 3). As duplicates show divergent expression patterns in both species, duplication potentially followed subfunctionalization of the copies under homogeneous purifying selection (see Results).

### (d) GPCR expansions and evolution of novel expression domains

In all expanded GPCR groups, we found evidence of tandem co-localization of genes in *O. bimaculoides* yet with diverging expression profiles (e.g. Scaffold 69 694 in group 4, figure 3; electronic supplementary material, table S2). Some of these were also observed to be in monophyletic groups with other non-co-localized genes (e.g. clade containing the genes of Scaffold 18 646 in group 1 or Scaffold 14 030 in group 2, figure 3). This indicates that the detected expression divergence could be a result of both accumulated mutations in the regulatory regions of clusters (for the case of co-localized genes, e.g. octopus-specific group 4) as well as the acquirement of new regulatory domains by rearrangement of gene order via translocation, gene conversion, or duplication and subsequent loss (e.g. cephalopod-specific groups 1 and 2) [63]. We found, additionally, significant signatures for positive selection on divergently expressed duplicates (e.g. groups 1 and 2, figure 3), suggesting an adaptive scenario for their retention and neo- or subfunctionalization.

Taken together, our phylogenomic analyses suggest a possible scenario for GPCR evolution. First, the large, anciently expanded (in the metazoan or bilaterian ancestors) GPCR complement shows a largely neuronal expression domain in cephalopods (figure 4; electronic supplementary material, figures S7–S9), indicating a high selective pressure to maintain nervous system-related functions. Genes duplicated in the cephalopod lineage (i.e. cephalopod-specific expansions) diverged their expression patterns to tissues mainly outside of the brain (figure 4). Translocation or segmental duplication and subsequent loss likely rearranged the original order of these cephalopod-specific duplicates and facilitated the evolution of novel expression domains. Some duplicates remained spatially linked, in which case accumulated mutations in their regulatory regions possibly coincided with the expression divergence between them. Finally, local gene duplications occurred more recently in the Octopodiformes lineage and resulted in the emergence of expression domains devoted to the axial nerve cord and associated structures (i.e. skin and suckers) in *O. bimaculoides* (figure 4; electronic supplementary material, figure S8), and to the gills, skin, and ovary in *C. minor* (figure 4; electronic supplementary material, figure S9). Here, in the case of physically co-localized genes with similar expression profiles, their expression patterns suggest a general regulatory property of those clusters (i.e. shared regulatory elements due to proximity [63] or tandem duplication of promoters and/or regulatory elements). Additionally, the GPCR expansions were accompanied by positive selection, suggesting functional adaptation during their evolution.

## 5. Conclusion

Duplication and divergence are powerful evolutionary mechanisms that can generate novel functions [10]. Lineage-specific GPCR expansions have predominantly occurred through this mechanism of gene duplication and subsequent independent evolution of the copies [5]. Here, we presented an analysis of the evolutionary history of the cephalopod GPCR repertoire. Our phylogenetic analysis distinguished cephalopod- and octopus-specific expansions from evolutionary older GPCR families (figure 1). Transcriptome data combined with genomic location of genes helped elucidate the likely evolutionary transitions between these expansions (figure 4). Our results reveal functional transitions in the evolution of cephalopod signal transduction, starting with non-expanded receptors having nervous system-related functions. Duplications shared between cephalopod lineages followed, which developed diverging expression across different tissues, in particular testes and gills. These were identified as neuropeptide receptors. Finally, more recent octopus-specific expansions with co-localized genes showed expression related to highly adapted octopod tissues and organs, such as the suckers and the axial nerve cord of the arms of octopuses (figures 3 and 4). These recent expansions were identified as Adhesion GPCRs and *methuselah/methuselah-like* GPCRs. These results help to reconstruct the evolutionary history of this superfamily of proteins and contribute to our understanding of the molecular mechanisms underlying the evolution of unique features in cephalopods.

## Supplementary Material

Supplementary Material

## Supplementary Material

Table S2

## Supplementary Material

Table S3

## Data accessibility

All supporting data (i.e. sequences, alignments, phylogenetic trees, expression matrices, Tau and expression proportion calculations, codeml files and likelihood ratio test calculations) are available from the Dryad Digital Repository: https://doi.org/10.5061/dryad.d3qh5c8 [37].
