# Supplementary material for: Genomic signatures of G-protein-coupled receptor expansions reveal functional transitions in the evolution of cephalopod signal transduction
Source: Proc Biol Sci. 2019 Feb 27;286(1897):20182929. doi: 10.1098/rspb.2018.2929 (PMC6408891; doi:10.1098/rspb.2018.2929)
Supplement: Supplementary Material [file rspb20182929supp1.docx]

**Supplementary Material**

**Genomic signatures of GPCR expansions reveal functional transitions in the evolution of cephalopod signal transduction**

Elena A. Ritschard^1,2^, Robert R. Fitak^2^, Oleg Simakov^1^, Sönke Johnsen^2^

^1^Department of Molecular Evolution and Development, University of Vienna. ^2^Duke University, Durham, NC, USA

Corresponding authors:

[elena.ritschard@univie.ac.at](mailto:elena.ritschard@univie.ac.at)

oleg.simakov@univie.ac.at

­­

1. **Figures**

**
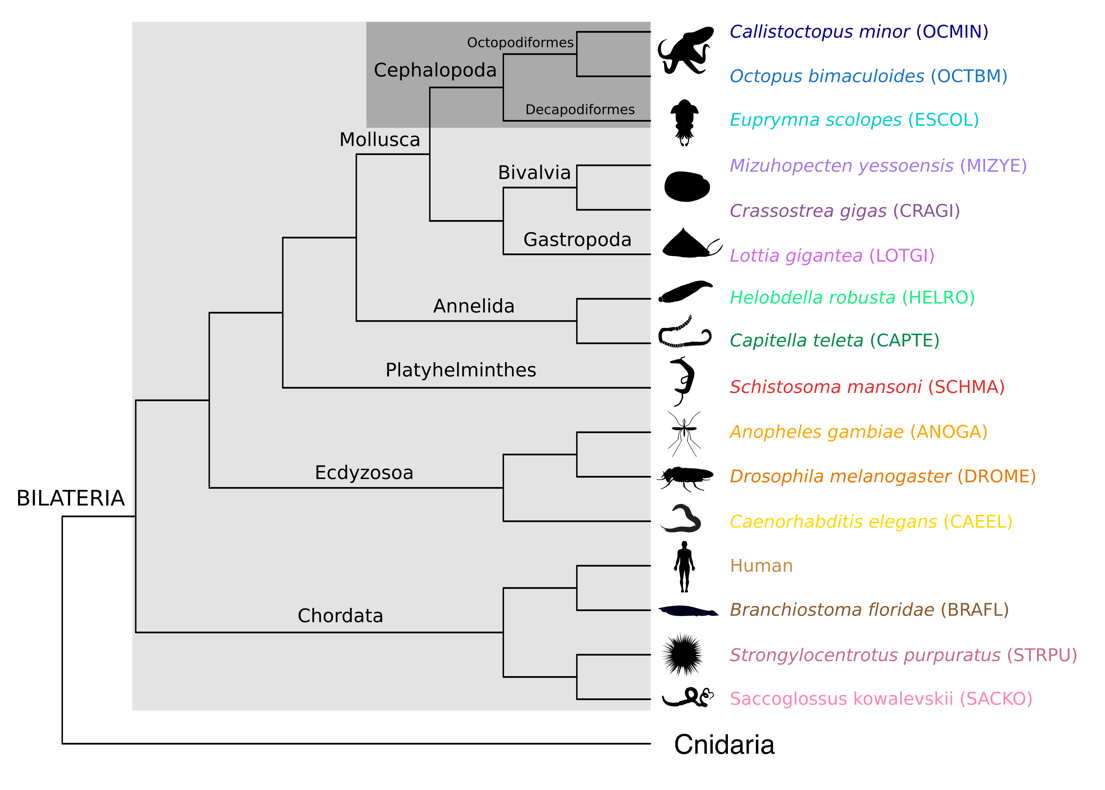
**

**Figure S1. Phylogeny of the taxa sampled.** GPCR repertoires from 16 bilaterian species here shown were used in our phylogenetic analyses. See Methods for more details. Octopus and squid illustrations were designed by Hannah Schmidbaur, all others were downloaded from Phylopic (<http://phylopic.org>).

**
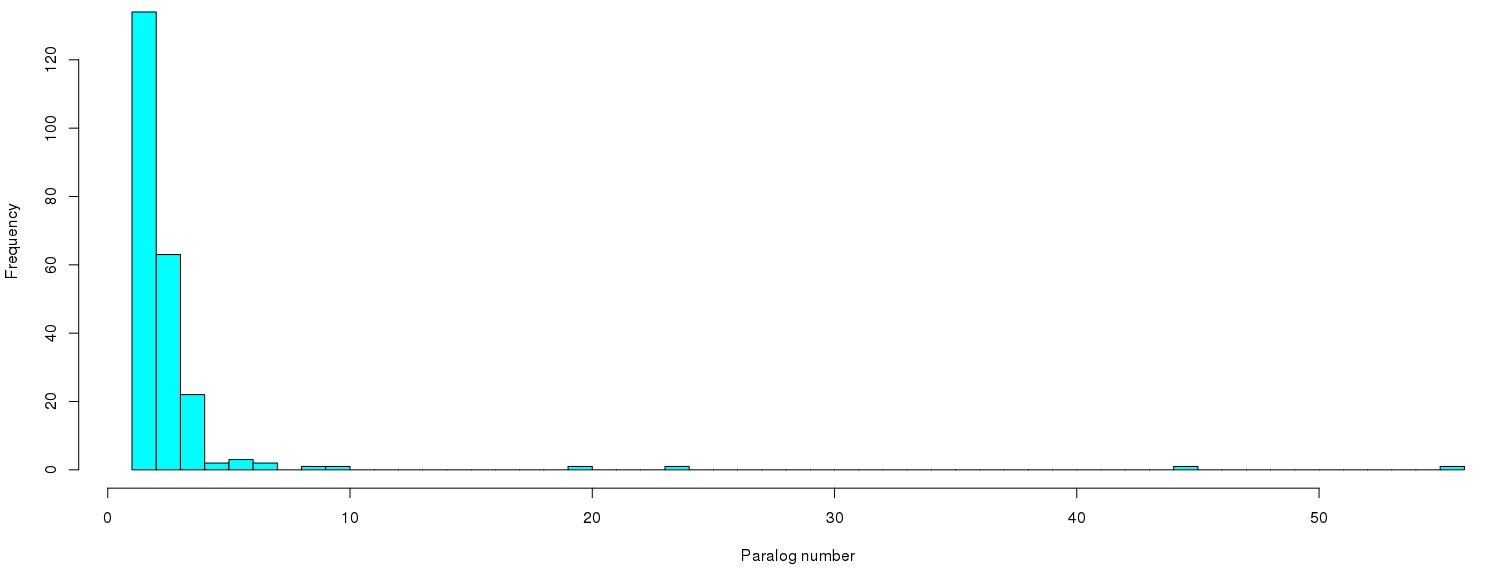
**

**Figure S2. Cut-off for the identification of expanded groups in our phylogenetic analysis.** The four bars with over 10 paralog sequences (*i.e.,* sequences belonging to one, two or the three cephalopod species used in the phylogenetic analysis) correspond to groups 1-4.


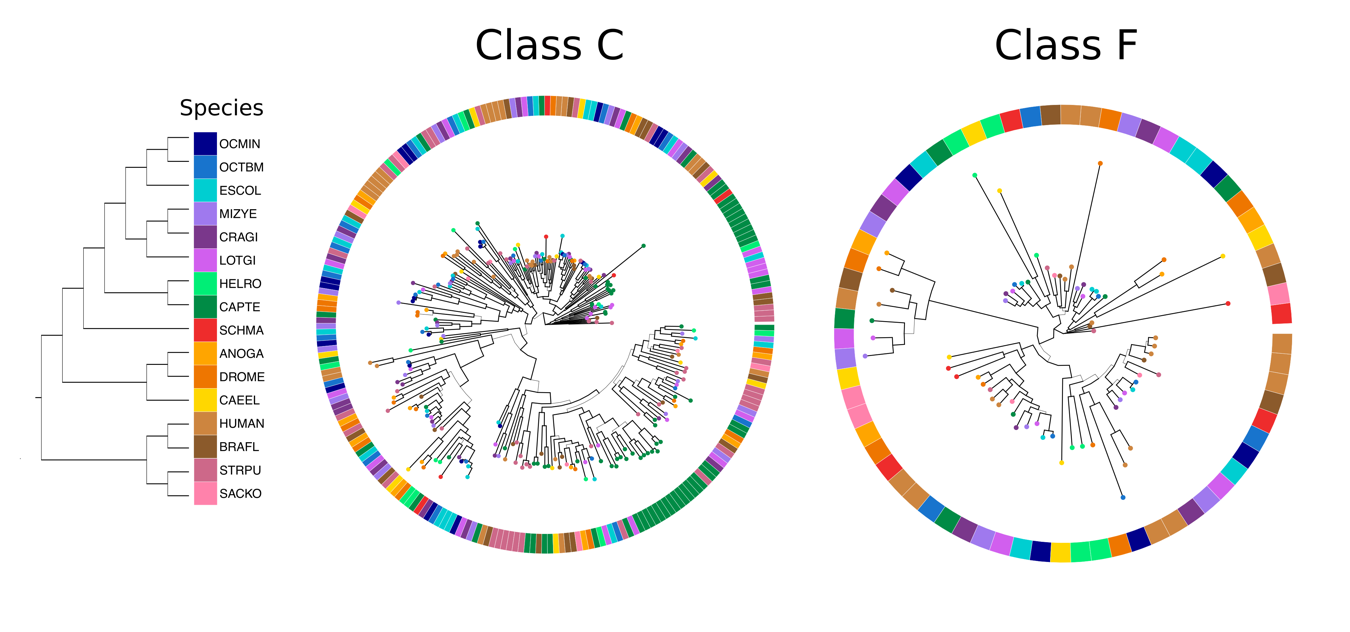


**Figure S3.** **Phylogenetic trees for class C and F.** Thicker branches represent significant S-H supports (>0.7) and each color corresponds to a species. ANOGA: *Anopheles gambiae,* BRAFL: *Branchiostoma floridae,* CAEEL: *Caenorhabditis elegans,* CAPTE: *Capitella teleta,* CRAGI: *Crassostrea gigas,* DROME: *Drosophila melanogaster,* ESCOL: *Euprymna scolopes,* HELRO: *Helobdella robusta,* HUMAN, LOTGI: *Lottia gigantea,* MIZYE: *Mizuhopecten yessoensis,* OCMIN: *Callistoctopus minor,* OCTBM: *Octopus bimaculoides,* SACKO: *Saccoglossus kowalevskii,* SCHMA: *Schistosoma mansoni,* STRPU: *Strongylocentrotus purpuratus.* No cephalopod expanded groups were found in these two GPCR classes.

**
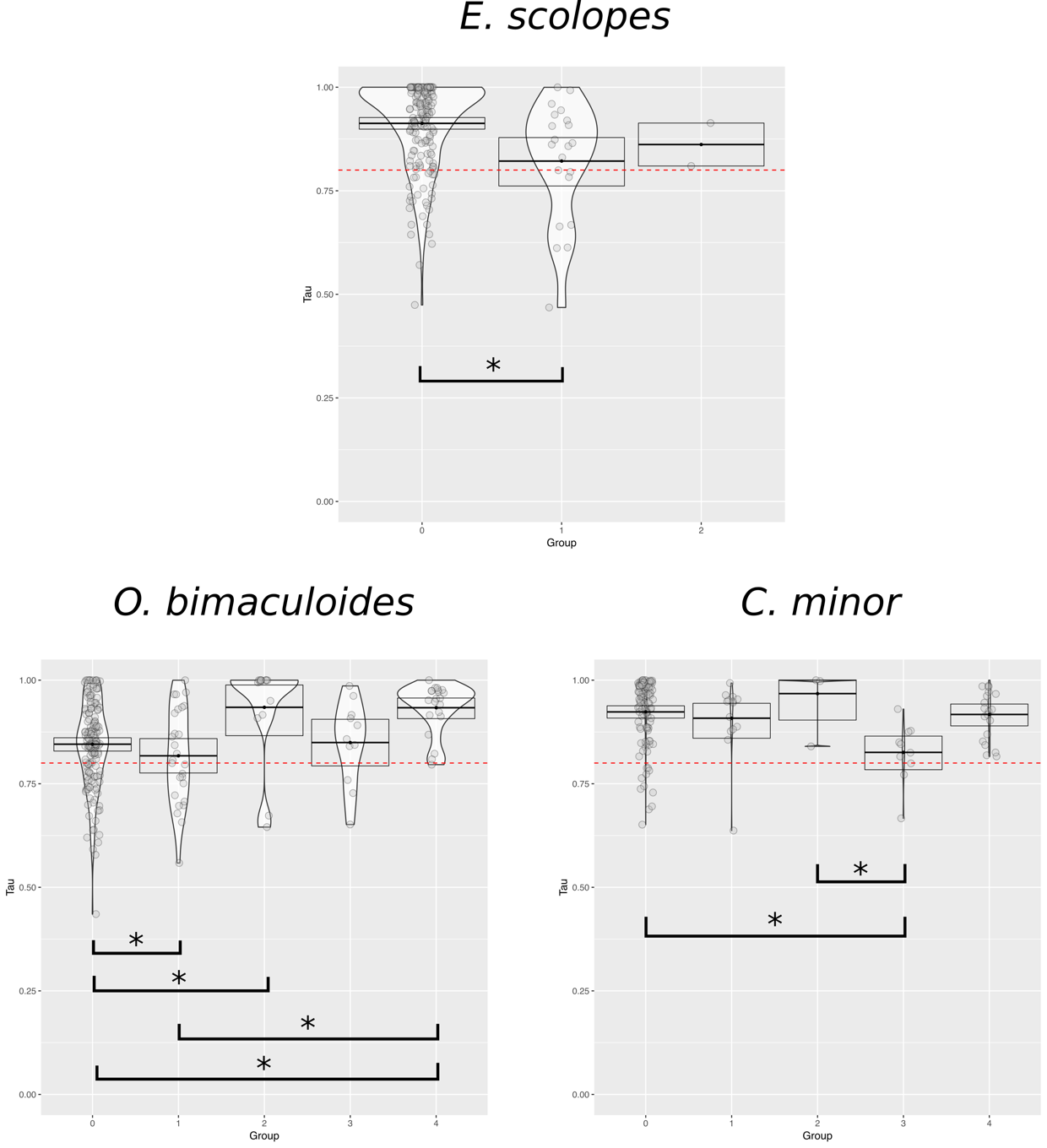
**

**Figure S4. Tau values in non-expanded GPCRS (0) and expanded groups 1-4 for *E. scolopes, O. bimaculoides* and *C. minor*.** Boxes represent the 95% confidence interval of the data distribution, black line in the middle the mean and the violin shape the density of data points (*i.e.,* more data points around a Tau value will generate a wider violin shape). The red dashed line represents the tissue-specificity threshold (Tau value 0.8). Bars with asterisks represent significant differences between the groups (Dunn’s test: p-value <0.025).


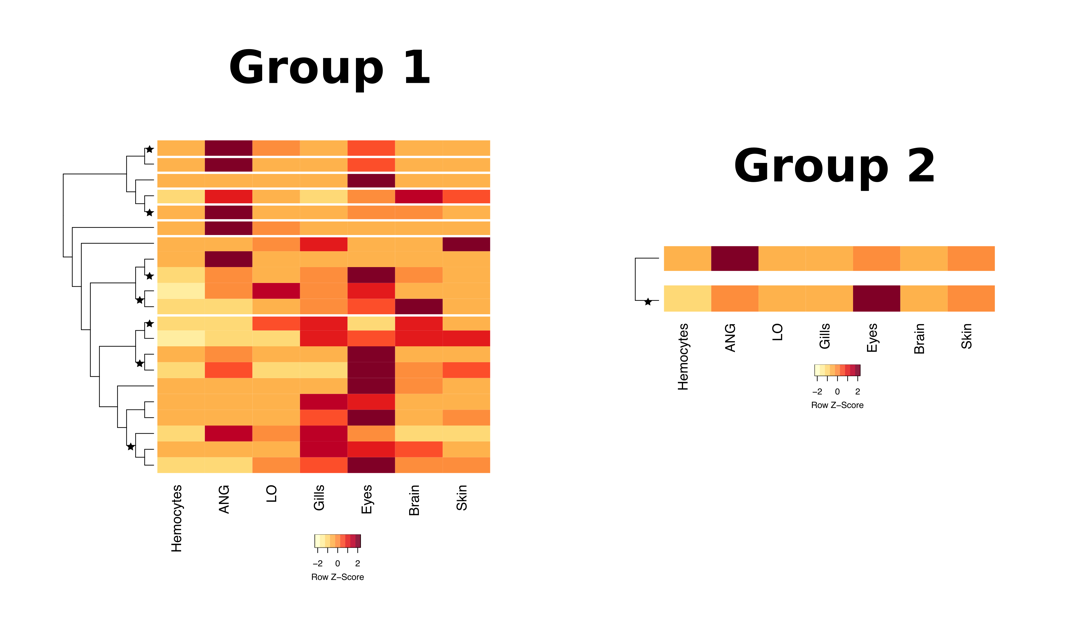


**Figure S5. Expression profiles and positive selection results cephalopod-specific expansions (groups 1 and 2) for *E. scolopes*.** Genes (rows) in heatmaps are clustered following the trees resulting from the phylogenetic analyses. Spaces between rows indicate the presence of other cephalopod species’ sequences as resulted in the phylogenetic analyses. Black stars represent positive selection (ω>1).


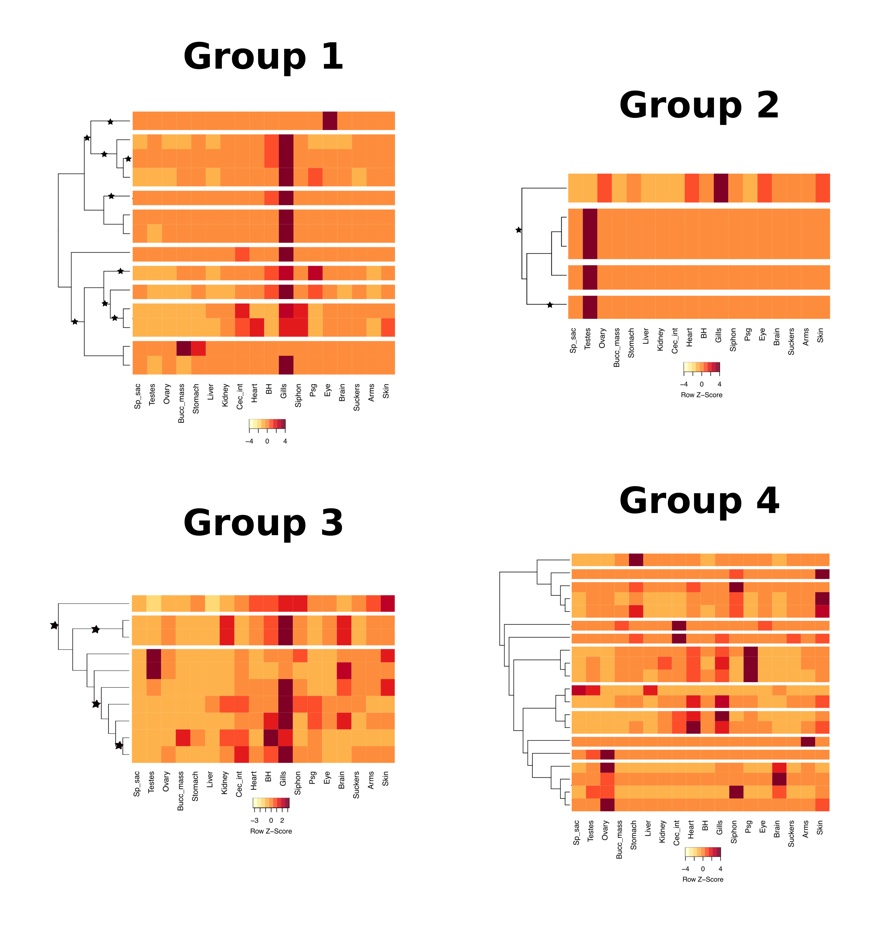


**Figure S6. Expression profiles and positive selection results cephalopod-specific expansions (groups 1 and 2) and octopus-specific expansions (groups 3 and 4) for *C. minor*.** Genes (rows) in heatmaps are clustered following the trees resulting from the phylogenetic analyses. Spaces between rows indicate the presence of other cephalopod species’ sequences as resulted in the phylogenetic analyses. Black stars represent positive selection (ω>1).


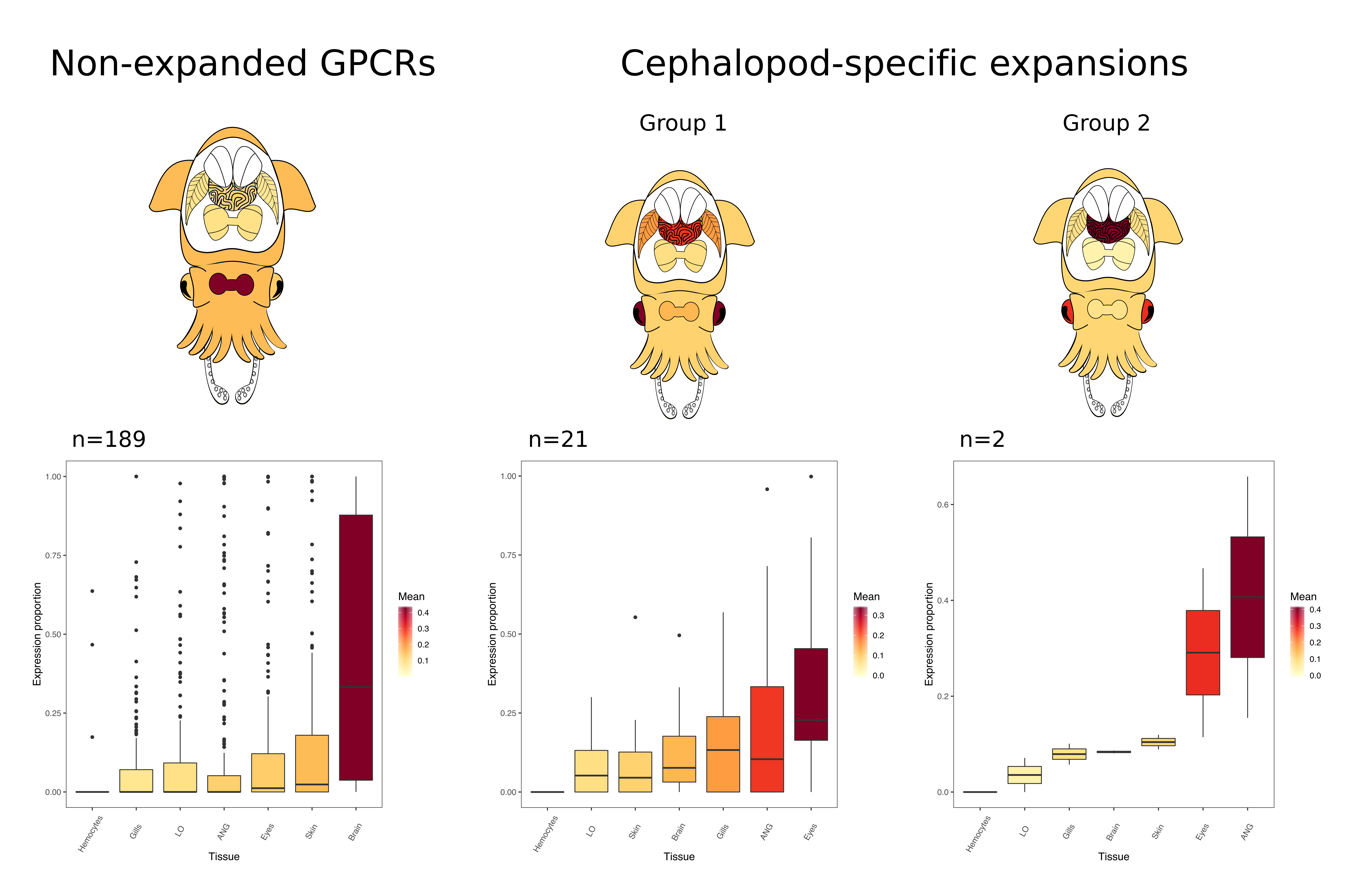


**Figure S7. Expression proportions ­­of *E. scolopes* genes in each tissue in non-expanded GPCRs and cephalopod-specific expansions.** Boxplots represent data between the first and third quartiles of the distribution and the median. Coloration gradient of boxplots follow the mean values of gene expression proportion in each tissue and was used to colour the corresponding tissues in the octopus’ diagrams above. Tissues: hemocytes, light organ (LO), accessory nidamental gland (ANG), skin, gills, brain and eyes. Number of data points in each boxplot: n.


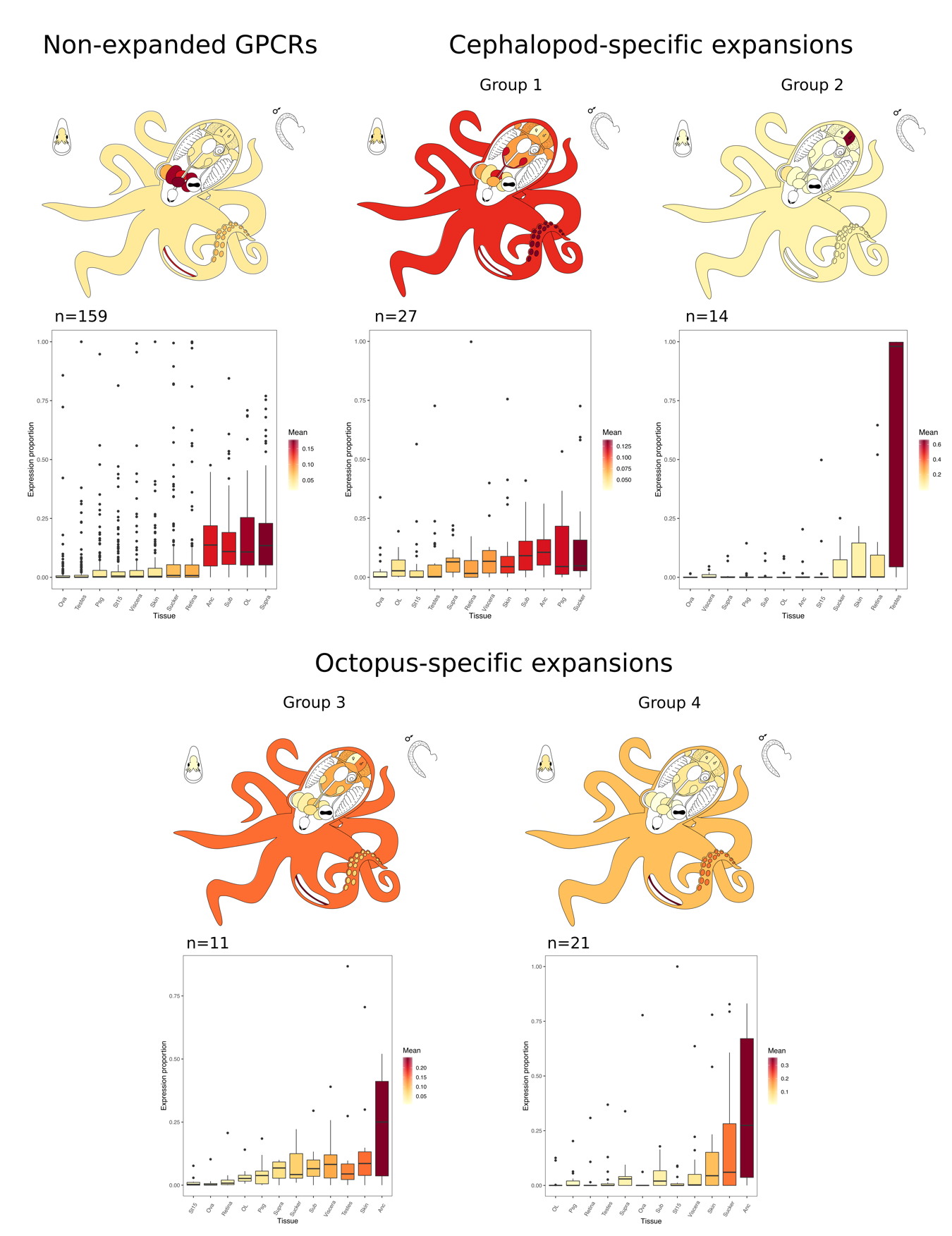


**Figure S8. Expression proportions ­­of *O. bimaculoides* genes in each tissue in non-expanded GPCRs, cephalopod-specific expansions and octopus-specific expansions.** Boxplots represent data between the first and third quartiles of the distribution and the median. Coloration gradient of boxplots follow the mean values of gene expression proportion in each tissue and was used to colour the corresponding tissues in the octopus’ diagrams above. Tissues: sucker, testes, stage 15 (St15) embryo, ova, skin, posterior salivary gland (Psg), viscera (heart, kidney and hepatopancreas), subesophageal brain (Sub), supraesophageal brain (Supra), optic lobe (OL), axial nerve cord (Anc) and retina. Number of data points in each boxplot: n.


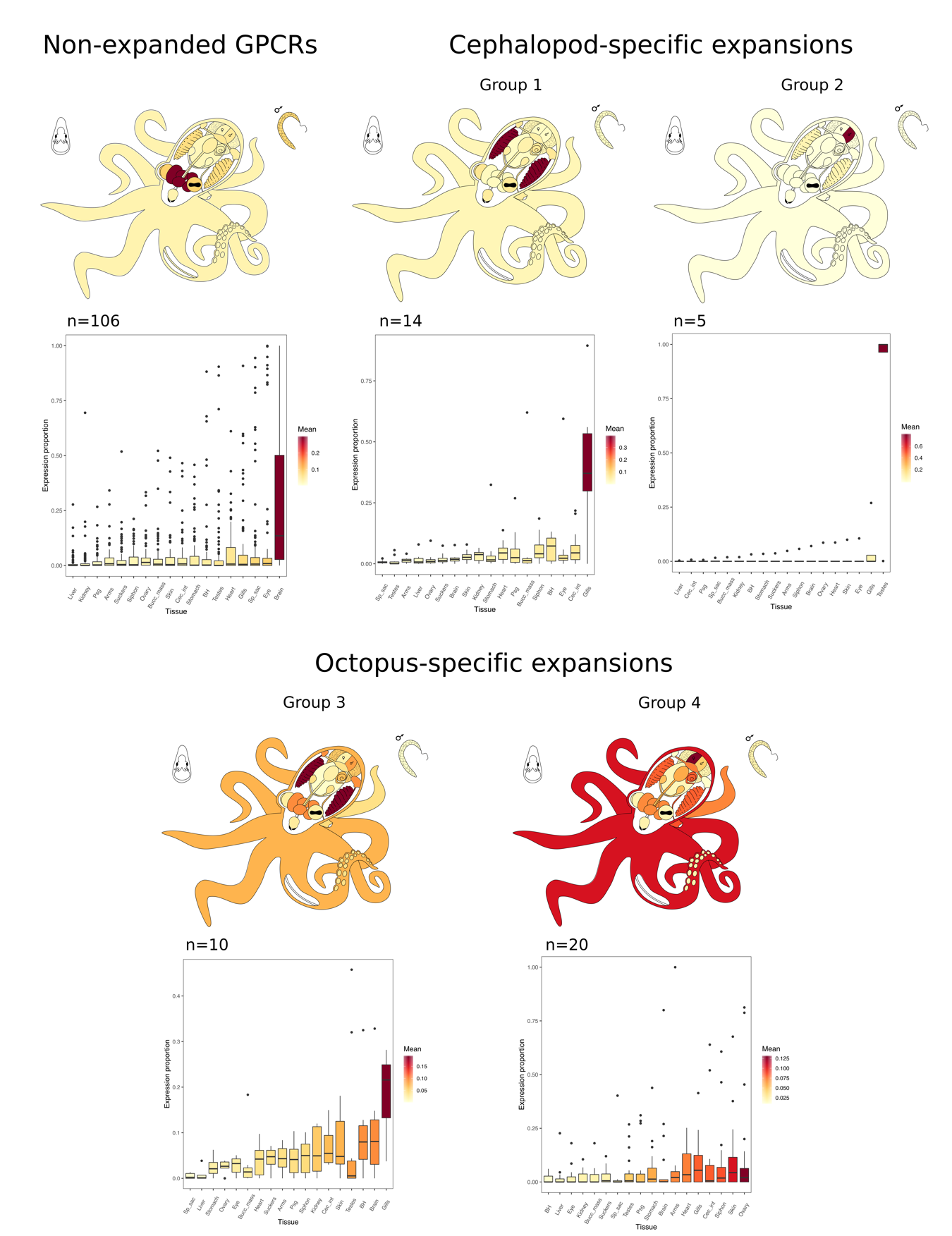


**Figure S9. Relative expression ­­of *C. minor* genes in each tissue in non-expanded GPCRs, cephalopod-specific expansions and octopus-specific expansions.** Boxes represent data between the first and third quartiles of the distribution and the black middle line the median. Coloration gradient of boxplots follow the mean values of gene expression proportion in each tissue and was used to colour the corresponding tissues in the octopus’ diagrams above. Tissues: liver, kidney, stomach, caecum intestine (Cec_int), posterior salivary gland (Psg), buccal mass (Bucc_mass), branchial heart (BH), systemic heart (heart), suckers, arms, skin, gills, siphon, brain, eye, spermatophore sac (Sp_sac), testes and ovary. Number of data points in each boxplot: n.

1. **Tables**

**Table S1. Summary of sequences used in the phylogenetic analyses.** The number of sequences is taken from final datasets after filtering duplicated gene entries (*i.e.,* isoforms).

| Species | Proteome entry | Number of sequences | | | | |
| --- | --- | --- | --- | --- | --- | --- |
|  |  | Class A | Class B | Class C | Class F | Total |
| Human | UP000005640 | 291 | 50 | 22 | 11 | 374 |
| *Branchiostoma floridae* | UP000001554 | 561 | 81 | 17 | 5 | 664 |
| *Strongylocentrotus purpuratus* | UP000007110 | 1019 | 213 | 32 | 5 | 1269 |
| *Saccoglossus kowalevskii* | - | 19 | 3 | 4 | 4 | 30 |
| *Caenorhabditis elegans* | UP000001940 | 129 | 5 | 8 | 4 | 146 |
| *Anopheles gambiae* | UP000007062 | 82 | 13 | 9 | 6 | 110 |
| *Drosophila melanogaster* | UP000000803 | 73 | 17 | 11 | 5 | 106 |
| *Schistosoma mansoni* | UP000008854 | 76 | 5 | 3 | 7 | 91 |
| *Helobdella robusta* | UP000015101 | 195 | 29 | 9 | 5 | 238 |
| *Capitella teleta* | UP000014760 | 935 | 39 | 51 | 5 | 1030 |
| *Lottia gigantea* | UP000030746 | 276 | 59 | 17 | 5 | 357 |
| *Crassostrea gigas* | UP000005408 | 362 | 63 | 14 | 4 | 443 |
| *Mizuhopecten yessoensis* | - | 357 | 46 | 15 | 5 | 423 |
| *Euprymna scolopes* | - | 232 | 39 | 19 | 7 | 297 |
| *Callistoctopus minor* | - | 190 | 66 | 19 | 2 | 277 |
| *Octopus bimaculoides* | UP000053454 | 233 | 81 | 19 | 6 | 339 |
| Total number of sequences | | 5030 | 809 | 269 | 86 | 6194 |

**Table S4. Summary of the positive selection analysis results.** Abbreviations: M0- null model, Ma-alternative model, np- number of parameters, df- degrees of freedom, LRT- Likelihood Ratio Test. Calculations can be found in “PositiveSelection_calculations.xlsx” in Dryad.

|  |  | Group 1 and 2 | Group 3 | Group 4 |
| --- | --- | --- | --- | --- |
| Branch-model test | M0 likelihood | -31390.1701 | -3636.4977 | -4193.2279 |
|  | M0 np | 281 | 59 | 119 |
|  | Ma likelihood | -30860.40443 | -3552.5782 | -4150.234 |
|  | Ma np | 435 | 96 | 202 |
|  | LRT | 1059.531342 | 167.839006 | 85.987776 |
|  | df | 154 | 37 | 83 |
|  | p-value | 0 | 0 | 3.89E-01 |
| Sites-model test | M0 likelihood | -31390.17 | -3636.4977 | -4193.2279 |
|  | M0 np | 281 | 59 | 119 |
|  | Ma likelihood | -31356.554 | -3566.5901 | -4153.9666 |
|  | Ma np | 284 | 62 | 122 |
|  | LRT | 67.23259 | 139.815336 | 78.522504 |
|  | df | 3 | 3 | 3 |
|  | p-value | 1.67E-14 | 0 | 1.11E-16 |

Tables S2 and S3 can be found as separate files of the Supplementary Material. Table captions as follow:

**Table S2. Summary of the expression analyses and gene locations.** Excel file contains spreadsheets with Tau and proportion of expression for each gene of 1) *Euprymna scolopes,* 2) *Octopus bimaculoides* and 3) *Callistoctopus minor*. Gene locations for *O. bimaculoides* are also shown here.

**Table S3. Summary of the annotation results.** Excel file contains spreadsheets with 1) InterProScan matches of the sequences of groups 1-4 for *Euprymna scolopes*, *Octopus bimaculoides* and *Callistoctopus minor* and 2) UniProt database descriptions of the nearest sequences to the expanded groups (1-4).
